# Evolution of Human Pair Bonds as a Consequence of Male-Biased Mating Sex Ratios?

**DOI:** 10.1007/s11538-025-01414-4

**Published:** 2025-01-30

**Authors:** Matthew C. Nitschke, Viney Kumar, Katrina E. Milliner, Kristen Hawkes, Peter S. Kim

**Affiliations:** 1 School of Mathematics and Statistics, The University of Sydney, Sydney, NSW 2006 Australia; 2 Department of Anthropology, University of Utah, Salt Lake City, UT 84112 USA

**Keywords:** Population dynamics, Mating strategies, Sexual conflict, Mate guarding, Human evolution

## Abstract

Compared to our closest primate relatives, human life history involves greater longevity, which includes a distinctive postmenopausal life stage. Given mammalian reproductive physiology in which females build a finite stock of cells that can become oocytes early in life, which then continuously deplete mostly through cell death while males produce new sperm throughout adulthood, the postmenopausal stage makes the sex ratio in the fertile pool, called the adult sex ratio (ASR), male biased. Additionally, this affects a more fine-grained ratio, the operational sex ratio (OSR), defined as the ratio of males to females currently able to conceive. Here, we construct an ODE model in which males compete for paternities using either a multiple-mating or mate-guarding strategy. Our focus is on investigating the differences of strategy choice between populations with varying life histories, which include a distinct post-fertile stage for adult females. By simulating the system, we determine the dominant strategy and its dependence on various parameter combinations. Our results show that an increase in OSR and ASR correlates well with a change in the dominant strategy from multiple mating to guarding.

## Introduction

The life history and social structure of mating behaviour differs considerably between humans and other primates. Although both humans and chimpanzees engage in a variety of mating strategies (Tutin 1979), it is only humans that form long-term preferential mating relationships (Muller and Pilbeam 2017). On reaching sexual maturity, the life histories of chimps and humans diverge. A chimpanzee at maturity may expect to live a further fifteen to twenty years (Hill et al. 2001; Muller and Wrangham 2014), but a human hunter-gatherer at the same point may expect to live for another forty years or more (Hill and Hurtado 2017; Howell 2017; Jones 2016). The age of last birth in both chimps and humans is at approximately 45 years. It is very rare for a chimpanzee to reach this age in the wild or captivity, but see Wood et al. (2017). However, it is common for hunter-gatherer females to live in good health past 70 years of age. Thus, those females who survive this long spend half of their adult lifespan as post-fertile. This causes a male-biased sex ratio where more males compete for paternities from a smaller number of fertile females. One of the prevailing hypotheses regarding the origin of monogamy in primates suggests that a male-biased sex ratio is the driving force behind its evolution (Coxworth et al. 2015; Schacht and Bell 2016).

Several other hypotheses have been proposed to explain the evolution and maintenance of monogamy (Klug 2018). However, more recent assessments have shown that these behaviours are more complex and some of the hypotheses describe the evolution of social organisation and/or social relationships and only have indirect implications for monogamy (Huck et al. 2020; Tecot et al. 2016). Thus, it is unlikely that there is a single theory that can incorporate all the factors that affect the emergence of these traits (Tecot et al. 2016). We briefly summarise several of these hypotheses.

The infant care hypothesis proposes that pair living and indirectly monogamous mating evolve when a mother requires the care of a male to successfully raise her offspring (Huck and Fernandez-Duque 2013; Kleiman 1977; Macdonald et al. 2019; Rogers et al. 2018; Schacht et al. 2018). Alternatively, the optimal group size hypothesis suggests that the optimal adult group size is two, which is a consequence of conflicting factors: protection from predators and the extent of food competition (Terborgh and Janson 1986). This hypothesis pertains directly to pair living and only indirectly to pair bonding and monogamy. Another hypothesis, the resource defense hypothesis, asserts that when resources are highly dispersed, low in quality, or rare, male–female pairs represent the most stable survival strategy (Fernandez-Duque 2016; Rutberg 1983). This applies directly to pair living and depending on additional factors, may apply to pair bonding and monogamy.

The infanticide prevention hypothesis (PALOMBIT 2000; van Schaik and Kappeler 2003; Wolff and Macdonald 2004) asserts that groups of male–female pairs and sexual monogamy form as a result of pressure females have to socialise with a male who can protect her and her offspring from other infanticidal males. This view was further explored in two highly cited papers that focused on mammals in general (Lukas and Clutton-Brock 2013) and more specifically primates (Opie et al. 2013). This hypothesis relates more closely to pair living than to sexual monogamy, since one of the traditional assumptions is that females mate with multiple mates as a counter-strategy against infanticide, which has some observational support (Hrdy 1974, 1979; van Schaik and Janson 2000; Chakrabarti and Jhala 2019; Wolff and Macdonald 2004; Hrdy 2009).

The mate guarding hypothesis proposes that a male will have the highest reproductive success if he defends mating access to a female from competing males, thus effectively mating persistently with her (Emlen and Oring 1977; Tecot et al. 2016). With a male-biased sex ratio, the competing pool of males makes claiming a female and defending that claim the strategy that wins more paternities than leaving to compete for paternity opportunities elsewhere (Parker 1974; Schacht and Bell 2016). This hypothesis endeavours to explain the evolution of monogamous mating; however, its ability to explain pair living depends on the length and predictability of the breeding season (Tecot et al. 2016). Our objective here is to investigate the factors that led to the earliest appearance of mate-guarding in our own evolution–a shift from the ancestral multiple-mating strategy–and not specifically the evolution of monogamy. However, we believe that pair bonding is a natural consequence of mate guarding.

Several recent mathematical models investigate the mechanisms behind this hypothesis by exploring the links between mating strategies, sex ratios and partner availability (Loo et al. 2017a, b; Schacht and Bell 2016; Schacht et al. 2017; Rose et al. 2019). Two such ratios appear frequently in the literature, the adult sex ratio (ASR), defined as the ratio of males to females in the fertile ages (Schacht and Bell 2016), and the operational sex ratio (OSR), which counts only the subset of adults currently capable of conception (Emlen and Oring 1977). Each ratio captures a different aspect of male competition; higher OSR corresponds to more competitions for each paternity opportunity, and a higher ASR to more competitions for each fertile female.

One of the more recent such models was provided by Schacht and Bell (2016) who include three male strategies: mate-guarding, multiple-mating and parental care. Under broad assumptions, they show the large effect of the ASR on male strategies concluding that mate guarding, rather than paternal care, likely propelled the evolution of human monogamy. Loo et al. (2017a, 2017b) significantly extended this work by allowing for guarding inefficiency and pair-bond break-up. They construct a system of ordinary differential equations that allows for an investigation into the effects of changes to parameters on the long-term equilibria of these strategies. The work of both Schacht and Bell and Loo et al. together support the hypothesis that mate-guarding is one of the primary forces behind the evolution of pair bonding. However, these models only include populations of males. A more recent model builds on these results by including additional populations of females and offspring to explore the link between the ASR and dominant male strategies (Rose et al. 2019). They include populations of females and subsequent offspring associated with males employing a particular strategy. Here, we extend this model to include post-fertile females and reconsider the role of the OSR as a predictor of male mating strategies.

Using a set of ordinary differential equations, we focus our attention on the factors that led to the emergence of mate-guarding behaviour. For simplicity, the only aspect of male-male or female–female competition included is chance and female choice is disregarded. Although both humans and chimpanzees exhibit a wide range of mating behaviours, our focus is on two strategies: mate guarding and multiple mating. We explore how changes in two life history features (1) the percentage of fertile males that increases as longevity expands, and (2) birth interval length which sets the frequency of paternity opportunities that affect the relative success of these male strategies. Shorter birth intervals mean more paternity chances as males have less time to wait for the next opportunity. This results in a lower OSR corresponding to less competition for each additional paternity. On the other hand, larger intervals require males to wait longer. This results in a higher OSR, corresponding to greater competition for each paternity. With an unbiased sex ratio, multiple-mating is the dominant strategy as waiting longer for the next opportunity takes away valuable opportunities elsewhere. Results of this study can be used to inform more detailed future models.

## Model

We construct a simple two-strategy ordinary differential equation (ODE) model, in which males either guard mates or multiply mate (possibly acquiring many paternities at nearly the same time), so there is a population *M* of multiple-mating males and a population *G* of guarding males searching for a mate. Females carry either the multiple-mating or guarding trait. When a female carrying the guarding trait mates with a guarding male, 100% of their offspring inherit this trait. Alternatively, if a female carrying the guarding trait mates with a multiple-mating male, half of their offspring inherit the guarding trait and the other half inherit the multiple-mating trait. When a guarding male finds a mate, he guards her, forming a pair bond. In contrast, a multiple-mating male continues competing for new mating opportunities. For simplicity, we assume males follow pure strategies of multiple mating or mate guarding throughout their lives and sons inherit their strategies from their fathers.

The model considers 17 populations: $F^M$, free females carrying the multiple-mating trait without dependants; $F^G$, free females carrying the guarding trait without dependants; *M*, multiple-mating males; *G*, unpaired guarding males; $F^M_m$, females with the multiple-mating trait caring for dependent offspring with the multiple-mating trait; $F^G_g$, females with the guarding trait caring for dependent offspring with the guarding trait; $F_m^G$, females with the guarding trait caring for a dependent offspring with the multiple-mating trait; $F_g^M$, females with the multiple-mating trait caring for dependent offspring with the guarding trait; $P_g^G$, pairs of guarding males and females with the guarding trait caring for dependants with the guarding trait; $P^G$, pairs of guarding males and females carrying the guarding trait without dependants; $P^M$, pairs of guarding males and females carrying the multiple-mating trait without dependants; $P^M_m$, pairs of guarding males and females with the multiple-mating trait caring for dependants with the multiple-mating trait; $P_g^M$, pairs of guarding males and females with the multiple-mating trait caring for dependants with the guarding trait; $P^M$, pairs of guarding males and females carrying the multiple-mating trait without dependants; *X*, post-fertile females without dependants; $X_m$, post-fertile females caring for dependent offspring with the multiple-mating trait; $X_g$, post-fertile females caring for dependent offspring with the guarding trait; and *Y*, retired males. We assume pair bonds can break up by death of either partner, at a constant break-up rate $\chi$, or by female fertility ending. We also assume males compete for additional mates up to an age of frailty. At this point, those in pairs continue reproducing with their mate until the pair bond ends, and those without a mate retire and enter the population of post-reproductive males, *Y*. If females are still caring dependants when a pair breaks up, they will continue to care for that dependant until maturity.

Our model is given by the ODE system (See Appendix C for a full description of each equation)

1 $$\begin{aligned} \frac{dF^M}{dt}&= -\rho F^M + \frac{1}{2} \beta (F_m^M +F_m^G +P_m^M+X_m)+(\beta + \delta _m) F_m^M +(\beta +\delta _g)F_g^M\nonumber \\&\quad + (\chi + \mu _M(t)) P^M - (\omega _F + \mu _F(t)) F^M, \end{aligned}$$

2 $$\begin{aligned} \frac{dF^G}{dt}&= -\rho F^G + \frac{1}{2} \beta (F_g^G + F_g^M +P_g^G+P_g^M+X_g)+(\beta + \delta _g) F_g^G + (\beta + \delta _m) F_m^G \nonumber \\&\quad + (\chi + \mu _M(t)) P^G - (\omega _F + \mu _F(t)) F^G, \end{aligned}$$

3 $$\begin{aligned} \frac{dM}{dt}&= \frac{1}{2}\beta (F_m^M+ F_m^G+P_m^M+X_m)+\frac{1}{2}q\beta (F_g^G+F_g^M+P_g^G++P_g^M+X_g)\nonumber \\&\quad -(\omega _M+\mu _M(t))M\,, \end{aligned}$$

4 $$\begin{aligned} \frac{dG}{dt}&= -\rho \left( \frac{G}{M+G}\right) (F^M+F^G)+\frac{1}{2}(1-q)\beta (F_g^G+F_g^M+P_g^G+P_g^M+X_g)\nonumber \\&\quad + (\chi +\omega _F+\mu _F(t))(P^M+P^G+P_g^M+P_g^G+P_m^M)-(\omega _M+\mu _M(t))G\,, \end{aligned}$$

5 $$\begin{aligned} \frac{dF_m^M}{dt}&= \rho \left( \frac{M}{M+G}\right) F^M+(\chi +\mu _M) P_m^M-(\beta +\delta _m+\omega _F+\mu _F(t))F_m^M\,, \end{aligned}$$

6 $$\begin{aligned} \frac{dF_g^G}{dt}&= \frac{1}{2}\rho \left( \frac{M}{M+G}\right) F^G+(\chi +\mu _M(t))P_g^G-(\beta +\delta _g+\omega _F+\mu _F(t))F_g^G\,, \end{aligned}$$

7 $$\begin{aligned} \frac{dF_m^G}{dt}&= \frac{1}{2}\rho \left( \frac{M}{M+G}\right) F^G-(\beta +\delta _m+\omega _F+\mu _F(t))F_m^G\,, \end{aligned}$$

8 $$\begin{aligned} \frac{dF_g^M}{dt}&=(\chi +\mu _M(t))P_g^M-(\beta +\delta _g+\omega _F+\mu _F(t))F_g^M\,, \end{aligned}$$

9 $$\begin{aligned} \frac{dP_g^G}{dt}&= \rho \left( \frac{G}{M+G}\right) F^G+\rho P^G-(\beta +\delta _g+\chi +\omega _F+\mu _M(t)+\mu _F(t))P_g^G\,, \end{aligned}$$

10 $$\begin{aligned} \frac{dP^G}{dt}&= -\rho P^G+(\beta +\delta _g)P_g^G-(\chi +\omega _F+\mu _M(t)+\mu _F(t))P^G\,, \end{aligned}$$

11 $$\begin{aligned} \frac{dP_m^M}{dt}&= \frac{1}{2}\rho \left( \frac{G}{M+G}\right) F^M+\frac{1}{2}\rho P^M-(\beta +\delta _m+\chi +\omega _F+\mu _M(t)+\mu _F(t))P_m^M\,, \end{aligned}$$

12 $$\begin{aligned} \frac{dP_g^M}{dt}&= \frac{1}{2}\rho \left( \frac{G}{M+G}\right) F^M+\frac{1}{2}\rho P^M-(\beta +\delta _g+\chi +\omega _F+\mu _M(t)+\mu _F(t))P_g^M\,, \end{aligned}$$

13 $$\begin{aligned} \frac{dP^M}{dt}&= -\rho P^M+(\beta +\delta _m)P_m^M+(\beta +\delta _g)P_g^M-(\chi +\omega _F+\mu _M(t)+\mu _F(t))P^M\,, \end{aligned}$$

14 $$\begin{aligned} \frac{dX}{dt}&= \omega _F(F^M+F^G+P^M+P^G)+(\beta +\delta _m)X_m+(\beta +\delta _g)X_g-\mu _F(t)X\,, \end{aligned}$$

15 $$\begin{aligned} \frac{dX_g}{dt}&= \omega _F(F_g^G+F_g^M+P_g^G+P_g^M)-(\beta +\delta _g)X_g-\mu _F(t)X_g\,, \end{aligned}$$

16 $$\begin{aligned} \frac{dX_m}{dt}&= \omega _F(F_m^M+F_m^G+P_m^M)-(\beta +\delta _m)X_m-\mu _F(t)X_m\,, \end{aligned}$$

17 $$\begin{aligned} \frac{dY}{dt}&= \omega _M(M+G)-\mu _M(t)Y\,. \end{aligned}$$

**Fig. 1:**
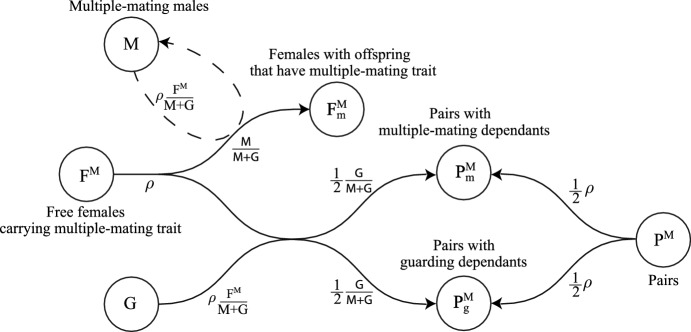
(Color Figure Online) Model diagram of mating interactions involving free females in $F^M$. Free females carrying the multiple-mating trait, $F^M$, productively mate at rate $\rho$. They mate with multiple-mating males, *M*, or unpaired guarding males, *G*, with probabilities $M/(M+G)$ or $G/(M+G)$. If they mate with multiple-mating males, they transition to the population $F_m^M$ of females caring for dependent offspring with the multiple-mating trait. If they mate with guarding males, they form pairs with the guarding males and together enter the population $P_m^M$ or $P_g^M$ with equal chance (thus the factor of 1/2). Paternity theft can occur only after the offspring matures. (Females drive productive mating at rate $\rho$, so all males, *M* and *G*, mate at rate $\rho F^M/(M+G)$, which is $\rho$ times the proportion of free females per unpaired male.) Pairs, $P^M$ of females and guarding males without dependants also productively mate at rate $\rho$ and enter population $P^M_m$ or $P^M_g$ with equal chance. Parameters and population variables are listed in Table 1

**Fig. 2:**
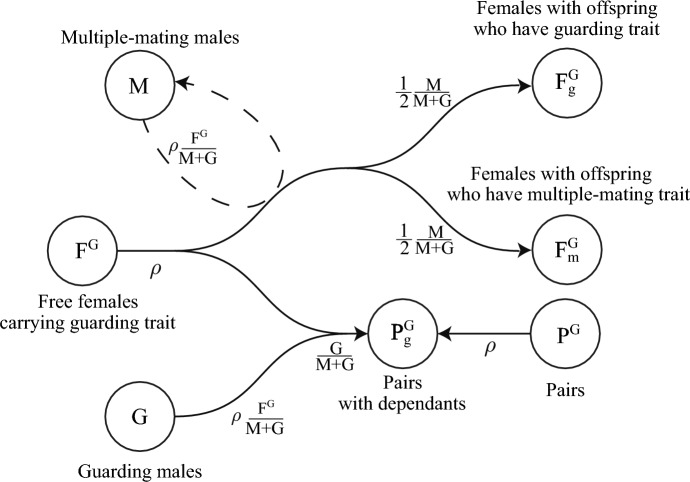
(Color Figure Online) Model diagram of mating interactions involving free females in $F^G$. Free females carrying the guarding trait, $F^G$, productively mate at rate $\rho$. They mate with multiple-mating males, *M*, or unpaired guarding males, *G*, with probabilities $M/(M+G)$ or $G/(M+G)$. If they mate with multiple-mating males, they transition to population $F_g^G$ or $F_m^G$ with equal probability (thus the factor of 1/2). If they mate with guarding males, they form pairs with the guarding males and together enter the population $P_g^G$. Pairs, $P^G$, of females and guarding males without dependants also productively mate at rate $\rho$ and enter population $P_g^G$.(Females drive productive mating at rate $\rho$, so all males, *M* and *G*, mate at rate $\rho F^G/(M+G)$, which is $\rho$ times the proportion of free females per unpaired male.) We include the chance that paternities from paired males can be stolen with probability *q*. In our model, this can only occur after pairs are established and the first successful conception by a guarding male. Parameters and population variables are listed in Table 1

**Fig. 3:**
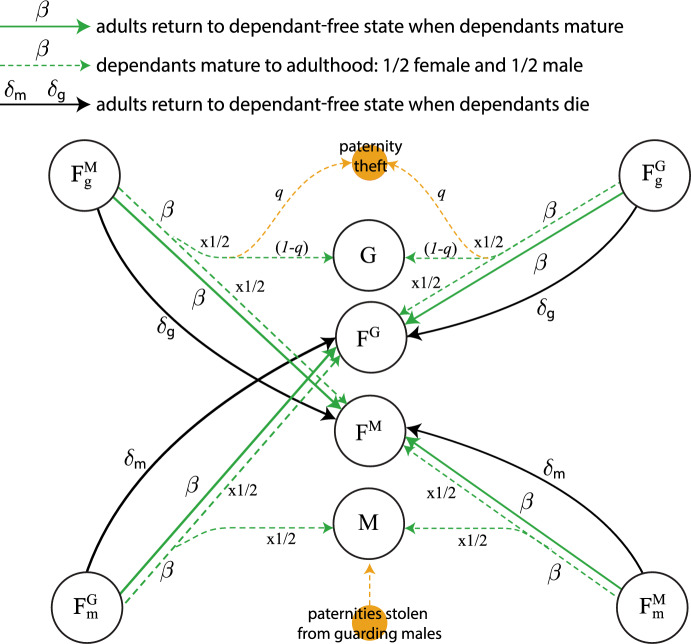
(Color Figure Online) Model diagram of dependant maturation to independence and death for unpaired females. When dependants mature, the adult carers return to the dependant-free state, and half of the maturing dependants enter one of the free female populations, $F^M$ or $F^G$, and the other half enter one of the male populations, *M* or *G*. Adult carers in the $F_g^i$, $F_m^i$ $(i=M,G)$ populations return to the dependent-free populations $F^i$ $(i=M,G)$. Also, maturing dependants from the $F_g^i$ $(i=M,G)$ populations divide half and half between the $F^i$ $(i=M,G)$ and *G* populations, unless the paternties are stolen by multiple-mating males with probability *q*. Similarly, maturing dependants from the $F_m^i$ $(i=M,G)$ populations divide half and half between the $F^i$ $(i=M,G)$ and *M* populations. When dependants die, adult carers return to the dependant-free state, and there are no maturing dependants to enter the adult populations. Parameters and population variables are listed in Table 1

**Fig. 4:**
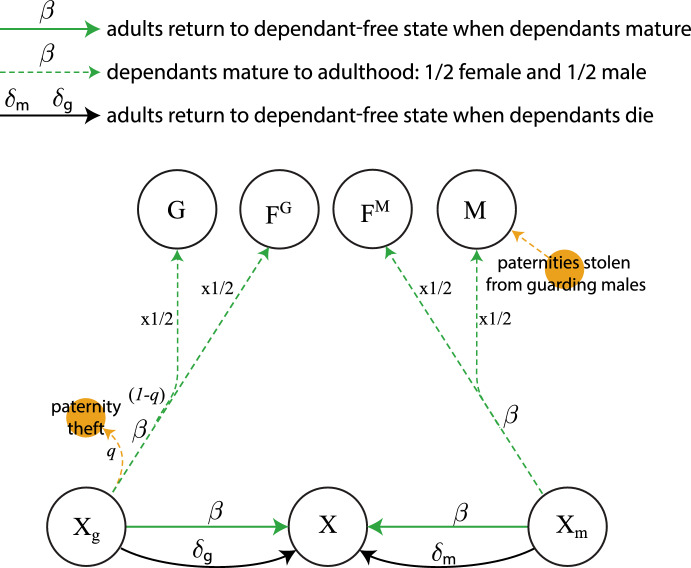
(Color Figure Online) Model diagram of dependant maturation to independence and death for unfertile females. When dependants mature, the adult carers in the $X_i$ $(i=m,g)$ populations return to the dependant-free population *X*. Also, maturing dependants from the $X_g$ population divide half and half between the $F^G$ and *G* populations, unless paternities are stolen by multiple-mating males with probability *q*. Similarly, maturing dependants from the $X_m$ population divide half and half between the $F^M$ and *M* populations. When dependants die, adult carers return to the dependent-free state, and there are no maturing dependants to enter the adult populations. Parameters and population variables are listed in Table 1

**Fig. 5:**
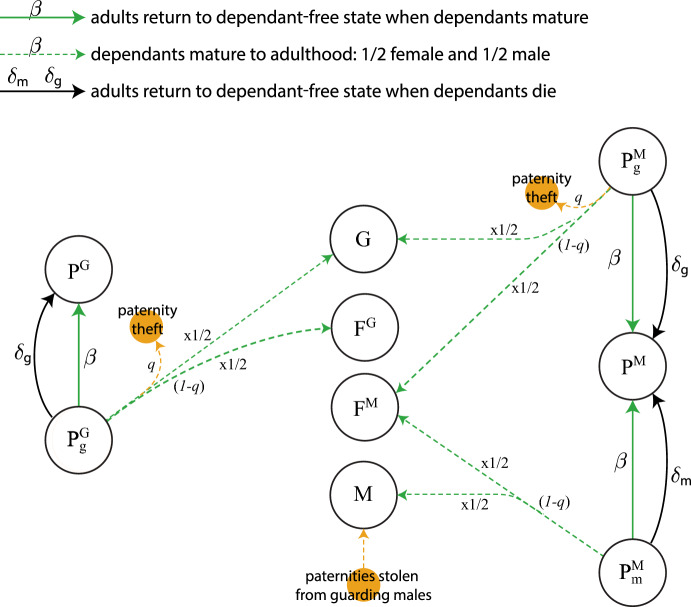
(Color Figure Online) Model diagram of dependant maturation and death for paired males and females. When dependants mature, the adult carers in the $P_g^G$, $P_g^M$, and $P_m^M$ populations return to the dependant-free populations $P^G$ and $P^M$ respectively. Also, maturing dependants from the $P_g^G$ and $P_g^M$ populations divide half and half between the $F^G$ and *G* populations, and maturing dependants from the $P_m^M$ population divid half and half between the $F^M$ and *M* populations, unless the paternities are stolen by multiple-mating males with probability *q*. When dependants die, adult carers return to the depandent-free state, and there are no maturing dependants to enter the adult populations. Parameters and population variables are listed in Table 1

**Fig. 6:**
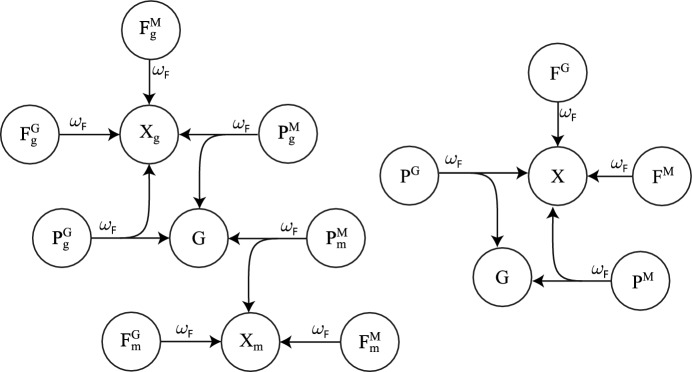
(Color Figure Online) Model diagram of the end of female fertility. Females lose fertility at rate $\omega _F$. When unpaired females in populations $F_g^i\,(i=M,G)$, $F_m^i\,(i=M,G)$, and $F^i\,(i=M,G)$ lose fertility, they transition to the post-fertile populations $X_g$, $X_m$, and *X*. When females in one of the paired populations $P^i_g\,(i=M,G)$, $P^i\,(i=M,G)$, or $P_m^M$ lose fertility, the pairs split up, causing the males to enter the unpaired guarding male population, *G*, and females to enter $X_g$, *X*, or $X_m$ respectively. Parameters and population variables are listed in Table 1

**Fig. 7:**
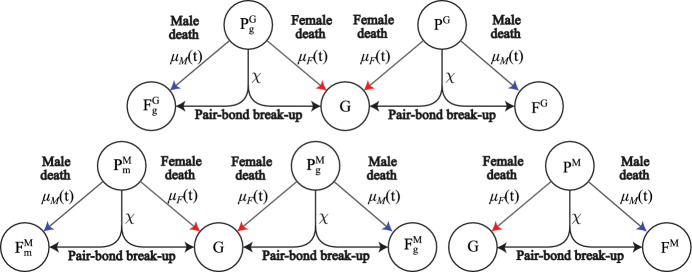
(Color Figure Online) Model diagram of break-ups and deaths of paired adults. When pairs $P_g^G$, $P^G$, $P_m^M$, $P_g^M$ or $P^M$ break up at rate $\chi$, the males return to the unpaired guarding population, *G*, and the females return to $F_g^G$, $F^G$, $F_m^M$, $F_g^M$ or $F^M$ depending on whether they have dependants and the traits they carry. When males in one of the paired populations $P_g^G$, $P^G$, $P_m^M$, $P^M$ die at rate $\mu _M(t)$, the remaining females return to $F_g^G$, $F^G$, $F_m^M$, $F_g^M$ or $F^M$ depending on whether they have dependants and the traits they carry. When females in one of the paired populations $P_g^G$, $P^G$, $P_m^M$, $P_g^M$, $P^M$ die at rate $\mu _F(t)$, the remaining males return to *G*. We also assume all unpaired adults die at rate $\mu _F(t)$ or $\mu _M(t)$, but these arrows are not shown to simplify the diagram. Parameters and population variables are listed in Table 1

Table 1 shows a summary of parameters and population variables. Figures 1, 2, 3, 4, 5 and 7 show model diagrams. Since the model interactions are complex, we divided them into seven separate diagrams. Figure 1 shows mating interactions with the $F^M$ population. Figure 2 shows mating interactions with the $F^G$ population. Figure 3 shows transitions due to dependant maturation and death with free female populations. Figure 4 shows transitions due to dependant maturation and death with non-fertile female populations. Figure 5 shows transitions due to dependant maturation and death with paired populations. Figure 6 shows transitions due to female fertility ending. Figure 7 shows transitions due to pair-bond break-ups and deaths of paired adults.

Multiple-mating males will mate with available females and remain active in the mating pool after each encounter. Females who mate with these males are unavailable (time-out) until their offspring are independent or die during care. Guarding males mate and form pair bonds with their mates. Both individuals in a pair then exclusively mate with each other until the bond breaks or the female becomes infertile at a constant rate. We have included the chance that there is paternity uncertainty, which is a measure of how effective the male is at keeping an eye on his female and preventing other males from mating with her. Multiple-mating males steal paternities from paired guarding males, since they are the ones who remain with their mates. We do not distinguish between guarding males, so guarders do not steal from each other in our model.

In (1)–(17), we use a female density-dependent death rate, $\mu _F(t)$, which contains two components:

18 $$\begin{aligned} \mu _F(t)&= \frac{1}{L}+\mu _{\text {variable}}(t), \end{aligned}$$

where the first term is a constant death rate given by the reciprocal of the individual’s expected adult lifespan *L* and the second term $\mu _{\text {variable}}(t) \ge 0$ is a variable density-dependent death rate, which serves to keep the population constant at its initial value, unless that would require the total death rate $\mu _F(t)$ to fall below the minimum death rate 1/*L*. In Appendix A, we derive the death rate

19 $$\begin{aligned} \mu _F(t) = \max \Big \{\frac{1}{L},\frac{\beta }{2}\Big (F_m^M+F_g^G+F_m^G+F_g^M+P_g^G+P_m^M+P_g^M+X_g+X_m\Big )\Big \}\,. \end{aligned}$$

In humans, as is common in mammals generally (Kruger and Nesse 2006; Lemaître et al. 2020), mortality is higher in males. Thus for males, we use the same density-dependent rate, but assume the baseline death rate differs by a small factor of 1.09, corresponding to a 9% higher minimum death rate. This is consistent with demographic data of hunter gatherers (Jones 2016). Hence, for males, we assume the death rate

20 $$\begin{aligned} \mu _M(t) = 1.09\cdot \mu _F(t)\,. \end{aligned}$$

Additionally, we investigate the effect of paternity theft, which occurs when a multiple-mating male steals the paternity from an unsuspecting guarding male. The probability of this occurring, *q*, depends on the proportion of multiple-maters in the adult male population. Thus, if we assume that $q^*$ is the success rate of multiple-mating thieves, then

21 $$\begin{aligned} q=\left( \frac{M}{M+G}\right) q^*\,. \end{aligned}$$

### Parameter Estimates

Conception times vary among great apes (Knott 2001), but human data suggests an average of 4 months, so we estimate a female conception rate of $\rho = 3$ yr$^{-1}$, corresponding to an average conception time of 1/3 yr, or 4 months (Gnoth et al. 2003). Estimating that on average, 65% of hunter-gatherer dependants survive to age four, and wild chimpanzees show a similar survival rate (Gurven and Kaplan 2007, Table 1), we obtain a dependant death rate of $\delta = -(1/4) \log (0.65) = 0.11$ yr$^{-1}$.

We estimate $\beta$, the rate dependants reach independence, using chimpanzee and human average interbirth intervals of 5.46 years and 3.69 years, respectively (Robson et al. 2006, Table 2.1). Rounding these values, we assume the interbirth intervals for chimpanzees and humans are 5 and 4 years and obtain the estimates $\beta = 1/5$ and $1/4 \text { yr}^{-1}$, but in our simulations, we also vary this parameter more widely to get a better sense of its effect. Note that independence for chimps and humans differs significantly. Unlike other ape infants who are feeding independently while still nursing, human infants are not capable of feeding on their own. Thus, our early weaning co-evolves with postmenopausal longevity.

Guarding could reduce dependant mortality by lowering the chance of infanticide by rival males. To investigate the possible effects of guarding, we define separate death rates $\delta _m$ and $\delta _g$ for offspring of multiple mating males and guarding males. Multiple mating offspring receive a disadvantage, *k*, which causes their death rate to increase relative to the fixed guarding offspring rate, $\delta _g$.

We include the probability *q* that fathers are uncertain of the validity of their paternities. As *q* increases, a larger proportion of youngsters cared by females paired to guarding males were sired by multiple-mating males and hence mature as multiple-mating males. In our simulations, we explore different values of this parameter, which represents guarding effectiveness.

To investigate the effect of pair-bond length on the dynamics of mating strategy, we also include the pair-bond breakup rate, $\chi$. A value of $\chi =1/n$ means that the average pair-bond lasts *n* years. Varying this parameter allows us to observe a greater range of strategies related to forming pairs.

Based on life-history regularities across primates, we assume key life-history transitions scale with respect to expected adult lifespans, *L* (Charnov et al. 1993). We assume females become sexually mature at age *L*/2. With this assumption, the age of female sexual maturity for chimpanzees and humans are $22/2 = 11$ and $38/2 = 19$, which fall near the ages of first birth in Robson et al. (2006). Then, we assume females reproduce up to age 45, which is close to the end of female fertility observed in both chimpanzees and humans (Hawkes 2010; Wood et al. 2023). The average female fertile years range from sexual maturity at *L*/2 to 45, yielding a total of $45 - L/2$ fertile years, so we estimate the average rate of female fertility loss to be the reciprocal

$$\begin{aligned} \omega _F = \frac{1}{45 - L/2} = \frac{2}{90 - L}. \end{aligned}$$

Note that this rate approximates the end of fertility, but female fecundity is constant during each female’s fertile years in our model. When a paired female’s fertility ends, her partner rejoins the mating pool and competes for another female to guard. Thus, there is no decline to the benefit of guarding that corresponds with fertility loss in our model. The case when there is no post-menopausal lifespan ($\omega =0$) is discussed in Appendix 2. In this case, it is shown that multiple-mating is always the dominant strategy.

We also assume males become less competitive with age and thus retire from actively seeking additional mates during their lifetime. Note that chimpanzee and human males have generally lower fertility rates at the end of their lifetimes (age $\sim 35$ in chimps and $\sim 60$ in hunter-gatherers (Jones 2016; Muller et al. 2020). This approximately scales with *L* when we observe that $60/38\approx 1.6$ for humans and $35/22\approx 1.6$ for chimpanzees. Thus, we estimate the average rate of male fertility loss to be the reciprocal

$$\begin{aligned} \omega _M=\frac{1}{1.6 L}. \end{aligned}$$

Parameter estimates are listed in Table 1.

**Table 1:** Parameters and population variables in (1)–(17)

Parameter	Description	Estimate (yr$^{-1}$)
$\rho$	Female conception rate	3
$\delta _m$	Death rate of multiple mating dependants	0.11*k*
$\delta _g$	Death rate of guarded dependants	0.11
*k*	relative guarding advantage multiplier	$1\le k \le 2$
$\beta$	Rate dependants leave mother	variable
*q*	Paternity uncertainty	(21)
$q^*$	Multiple mating theft success rate	$0 \le q^*\le 1$
$\chi$	Pair-bond break-up rate	variable
*L*	Expected adult lifespan	22 yr (chimp), 38 yr (human)
$\omega _F$	Rate of female fertility loss	0 (Case 1), $2/(90-L)$ (Case 2)
$\omega _M$	Rate of male fertility loss (frailty)	1/(1.6*L*)
$\mu _F(t)$	Female adult death rate	(19)
$\mu _M(t)$	Male adult death rate	(20)

Variable	Description	
$F^M$	Population of free females carrying the multiple-mating trait	
$F^G$	Population of free females carrying the guarding trait	
*M*	Population of multiple-mating males	
*G*	Population of unpaired guarding males	
$F_m^M$	Population of unpaired females with the multiple-mating trait caring for dependent offspring with the multiple-mating trait	
$F_g^G$	Population of unpaired females with the guarding trait caring for dependent offspring with the guarding trait	
$F_m^G$	Population of unpaired females with the guarding trait caring for dependent offspring with the multiple-mating trait	
$F_g^M$	Population of unpaired females with the multiple-mating trait caring for dependent offspring with the guarding trait	
$P_g^G$	Population of pairs of guarding males and females with the guarding trait caring for dependants with the guarding trait	
$P^G$	Population of pairs of guarding males and females carrying the guarding trait without dependants	
$P_m^M$	Population of pairs of guarding males and females with the multiple-mating trait caring for dependent offspring with the multiple-mating trait	
$P_g^M$	Population of pairs of guarding males and females with the multiple-mating trait caring for dependent offspring with the guarding trait	
$P^M$	Population of pairs of guarding males and females carrying the multiple-mating trait without dependants	
*X*	Population of post-fertile females without dependants	
$X_m$	Population of post-fertile females caring for dependent offspring with the multiple-mating trait	
$X_g$	Population of post-fertile females caring for dependent offspring with the guarding trait	
*Y*	Population of retired males without a mate and no longer competing for paternities	

For the rate of female fertility loss, Case 1 corresponds to female fertility not ending and hence no post-menopausal lifespan, and Case 2 corresponds to female fertility ending at age 45, which leads to an increased human post-menopausal lifespan

## Results

Our goal is to investigate populations where males employ two possible mating strategies: multiple-mating and guarding. We do this by exploring the parameter space of the ODE model described in the previous section. More precisely, we wish to determine the dominant strategy at equilibrium corresponding to a particular set of parameters.

**Fig. 8:**
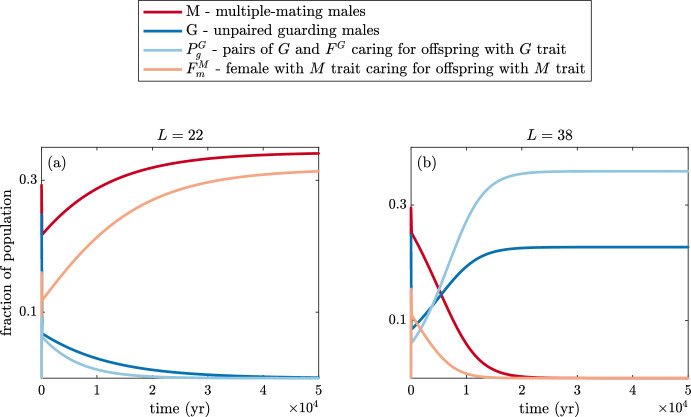
(Color Figure Online) Time evolution of the model for various populations. Solutions are shown when *L* is **a** 22 and **b** 38. Multiple mating is the only surviving strategy when the population has a shorter average lifespan of 22 years. Alternatively, when the average lifespan is longer, only guarding individuals survive. With a longer average lifespan, more females survive past their reproductive years, creating a male-biased population. All parameters are as in Table 1 with no paternity uncertainty and no pair-bond break up ($q=0$, $\chi =0$). Initial conditions for all cases are $F^M(0)=0.5$, $F^G(0)=0.5$, $M(0)=0.5$, $G(0)=0.5$ with all other populations starting at 0. Note that for almost all parameter combinations, the solutions look like one of these figures with one strategy completely dominant at equilibrium and the other extinct

To provide a baseline from which to compare parameters, we start by setting the paternity uncertainty, *q*, and pair-bond break-up rate, $\chi$, equal to zero. Trajectories of solutions with these assumptions are shown in Fig. 8 for populations with parameters found in Table 1.

Note that when mean lifespan increases from $L=22$ to $L=38$, there are more unpaired, still-fertile males competing for paternities. When the population has a longer average lifespan, more females survive beyond their reproductive years (female fertility ends at 45), creating a male-biased population. In the next section, we analyse these results over parameter space and estimate both the ASR and OSR.

### Male-Biased Sex Ratios

We start our analysis by observing the parameter space and the strategy that dominates each point of that space. To do this, we generate a grid of points in parameter space for a range of parameter combinations. In each case, we track males who employ the multiple-mating and mate-guarding strategies. The strategy that results in a surviving population at equilibrium is the successful strategy and the corresponding colour is filled in. Note that except for a few cases at the boundary between regions, the simulation results in survival for only one strategy. Unless otherwise noted, all of the plots allow for a non-zero rate of fertility loss, corresponding to Case 2 in Table 1. Initial conditions were kept the same for each set of parameters: $F^M(0)=0.5$, $F^G(0)=0.5$, $M(0)=0.5$, $G(0)=0.5$, and all other populations starting at zero. However, the system only has one non-trivial equilibrium so the choice does not alter the equilibrium attained. Additionally, both the ASR and OSR (as defined in Table 2) are determined for the model at equilibrium. These ratios are included to illustrate the underlying dynamics of the population and to predict the transition between regions where one strategy dominates.

**Table 2:** Definition of ASR and OSR with variables in Eqs. 1–17

Ratio	Definition
Adult sex ratio	$\displaystyle \frac{M+G+P_g^G+P^G+P_m^M+P_g^M+P^M}{F^M+F^G+F_m^G+F_m^M+F_g^G+F_g^M+P_g^G+P_g^M+P^G+P_m^M+P^M}$
Operational sex ratio	$\displaystyle \frac{M+G}{F^M+F^G}$

Population variables are as in Table 1

**Fig. 9:**
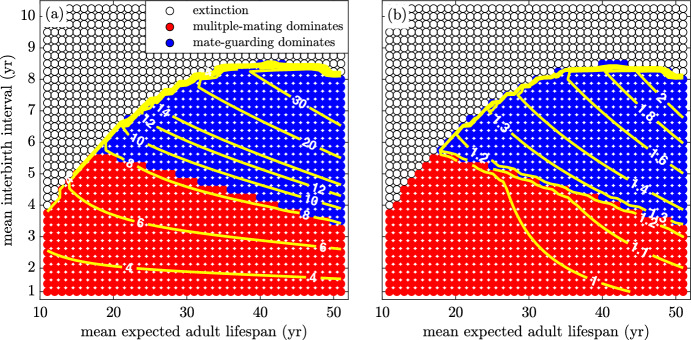
(Color Figure Online) Dominant strategy when interbirth interval and mean expected adult lifespan are varied. Contour lines show constant OSR and ASR. **a** The contour lines of constant OSR and **b** ASR provide a good indication of where the strategy shift is likely to take place. At each point on the figure, except for a few points at the boundary between regions, the dominant strategy is the only surviving population. Parameters and initial conditions are the same as in Fig. 8

A comparison of the OSR and ASR when the paternity uncertainty and pair-bond break-up rate are zero is illustrated in Fig. 9. Both ratios provide a good indication of where the strategy shift is likely to occur. As both ratios increase, there is greater competition among males for each available female. These larger ratios are caused, in part, by longer interbirth interval lengths. When each offspring requires more years of care, females are out of action for a greater proportion of their fertility window. This effect is offset by a greater expected longevity which lengthens the fertility window, causing the contour lines to slope downward. The same figure for the case when female fertility does not end ($\omega =0$) for the same set of parameters is shown in the Supplementary Material. In this case, multiple mating is the only strategy that survives. Using these results, we can obtain insights into the mechanisms underlying the evolutionary shift in male mating strategies to long-term pair bonds.

**Fig. 10:**
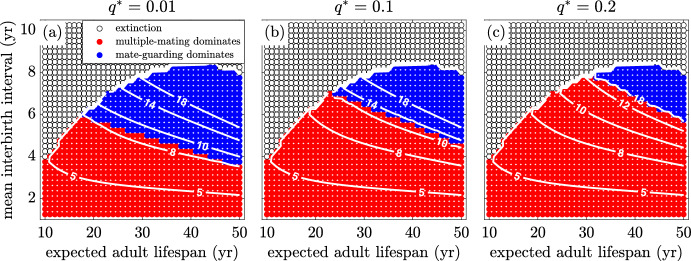
(Color Figure Online) The effect of paternity uncertainty. The success rate, $q^{*}$, of multiple-mating males is increased from **a** 1% to **b** 10% and **c** 20%, which results in a shrinking guarding region. All other parameters are as in Table 1

**Fig. 11:**
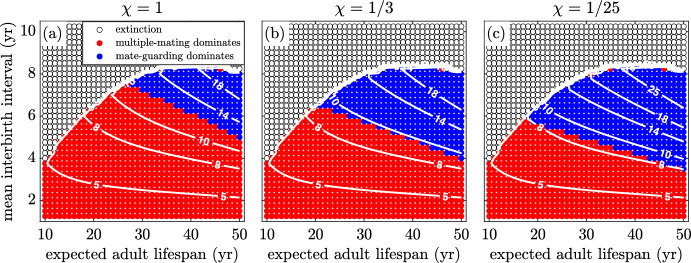
(Color Figure Online) The effect of pair break-up rate, $\chi$. As the pair bond break-up rate decreases from an average duration of **a** 1 year to **b** 5 years and **c** 25 years, the guarding region grows in size. Thus, the OSR at the boundary between regions decreases. For all other parameters, see Table 1)

**Fig. 12:**
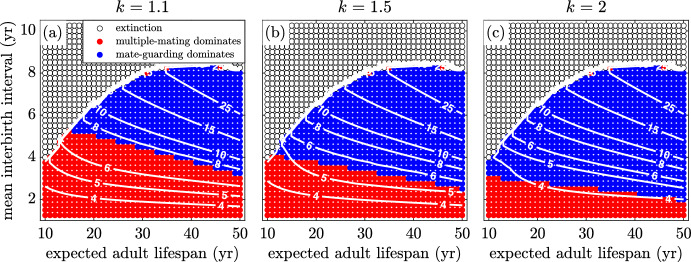
(Color Figure Online) The effect of changing dependant mortality. The death rate of multiple-mating dependants, $\delta _m$, is increased relative to that of guarded offspring, $\delta _g$. As $\delta _m$ is increased to **a** 10% **b** 50% and **c** 100% above $\delta _g$, the guarding region increases in size. Note that the OSR at the transition between regions becomes lower. (see Table 1)

We investigate the effects of increasing paternity uncertainty, *q*, pair-bond break-up rate, $\chi$, and relative dependant mortality in Figs. 10, 11 and 12. To do this, we start with the baseline model corresponding to Fig. 9 and vary *q*, $\chi$, and *k*. On each figure, contour lines of constant OSR are included that fall near the boundary between regions.

Figure 10a, b and c illustrate the result of increasing the paternity uncertainty, *q*. As expected, greater paternity uncertainty makes it less advantageous to invest effort into mate guarding. If there is a greater chance that multiple-mating males steal the paternity, the advantage of guarding rapidly declines. Thus, as paternity uncertainty increases, the mate-guarding region shrinks. An increasing paternity uncertainty means that the sex ratio must be more male biased for there to be sufficient incentive to favour guarding over multiple mating, thus the higher OSR contour lines at the boundary between regions.

In Fig. 11a, b and c we explore how the pair-bond break-up rate affects the system. When break-up is introduced and becomes more frequent, the mate-guarding region shrinks in size. This follows expected behaviour, since it is less advantageous to invest in mate guarding if the average pair bond is short-lived. Note that the OSR on the boundary of the region drops as pairs last longer. This implies that shorter pair duration requires the sex ratio to be more male biased for there to be sufficient selective pressure to favour guarding over multiple mating.

In Fig. 12a, b and c we investigate how a reduced dependant mortality may affect mating strategies. This does not explicitly model caring as a strategy, which was considered by others (Hawkes et al. 1995; Schacht and Bell 2016), but instead a beneficial consequence of increased guarding behaviour (guarding could reduce dependant mortality by lowering the chance of infanticide (van Schaik and Janson 2000; Hrdy 1974; 1979; 2009). If guarding reduces infant mortality by more than ten percent, the guarding region slightly increases in size. As this mortality rate is further reduced, the OSR at the boundary of the two regions decreases. Thus, males need less incentive to guard as this behaviour not only requires less competition for each additional paternity, but also it provides for better potential survival of each success.

**Fig. 13:**
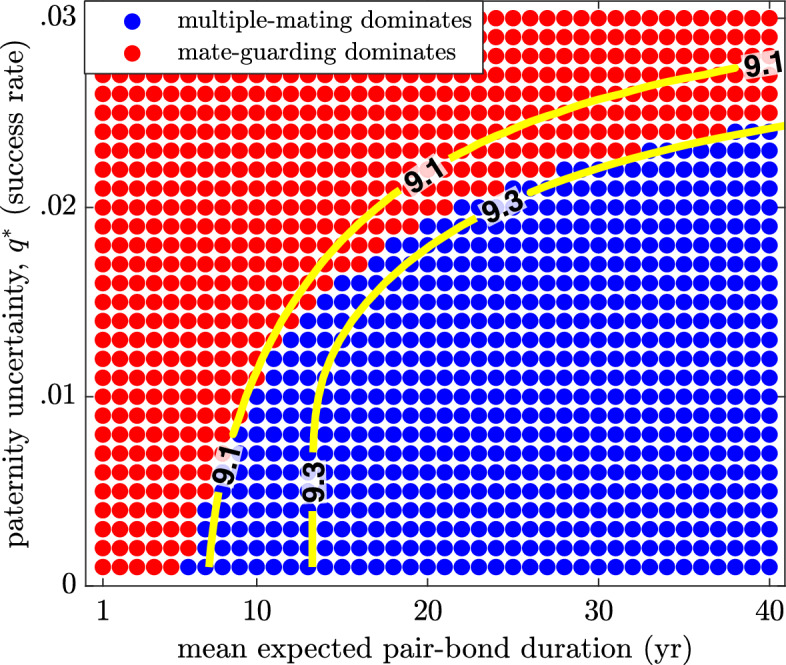
(Color Figure Online) Dominant strategies when paternity uncertainty (*q*) and mean expected pair-bond duration ($1/\chi$) vary in a population where expected longevity is 30 years and the interbirth interval is 5 years ($L=30$, $\beta =5$). Included are several lines of constant OSR, which provide an approximate indication of where the strategy shift is likely to take place. Parameters are as in Table 1

Another view that clarifies how these two parameters affect the dominant mating strategy is shown in Fig. 13. In this case, we fix a single point in the *L*-$\beta$ space corresponding to a population between chimps and humans for which $L=30$ and $\beta =5$. Then we vary paternity uncertainty, *q*, and average pair bond duration, $1/\chi$. We observe that a curve of constant OSR of between 9.1 and 9.3 roughly corresponds to the boundary between the regions where guarding or multiple mating dominate (approximately 9 males for each female). Increasing paternity uncertainty causes guarding incentive to drop as the expected payoff is reduced. In contrast, longer pair bonds cause an increase in guarding incentive, since this guarantees more paternity opportunities in the long run. However, there is a trade-off between these two parameters. If pair-bond duration is big enough, it can overcome a greater degree of paternity uncertainty as there will be more overall paternity opportunities, even if the uncertainty is higher.

**Fig. 14:**
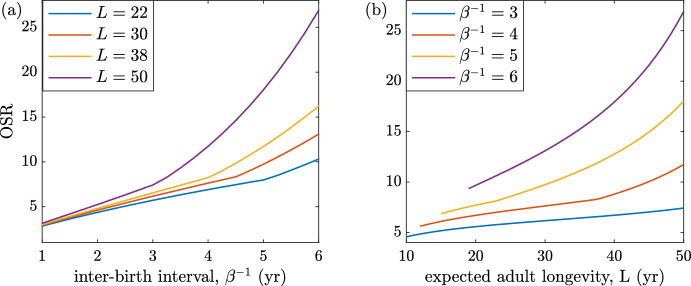
(Color Figure Online) Operational (OSR) sex ratio as a function of interbirth interval and longevity. **a** For several fixed *L* values, the OSR is shown for increasing interbirth intervals. The OSR displays an exponential increase as the interbirth interval increases in length. A lengthening interbirth interval keeps females out of the pool available for a conception longer and causes the population to grow slower over time. This results in a higher proportion of older, post-fertile females in the population. **b** For several fixed interbirth interval lengths ($\beta ^{-1}$), the OSR is shown for increasing *L* values.As both expected adult longevity and interbirth interval length increase, the OSR responds with an increase. Other parameters are as in Table 1 with $q=0$ and $\chi =0$

Figure 14a demonstrates how the OSR is affected by the interbirth interval for several fixed expected adult longevity values, *L*. Note that as the interbirth interval increases, the OSR increases. This occurs because as the birth interval increases, females are kept out of the pool available for conception longer and the population grows slower over time. As a result, fertile females are kept out of the mating pool for longer, increasing the male bias, and there is a higher proportion of older, post-fertile females. Since we assume males are fertile throughout their lives, this implies that the OSR will increase as a consequence. Another view is provided by Fig. 14b for several fixed interbirth intervals, $\beta ^{-1}$, and varying values of *L*. In a similar way, we see that as longevity increases and the end of female fertility remains fixed, the proportion of older, still-fertile males increases, leading to higher sex ratios. The sex ratios measure the competition that each male faces for a single paternity opportunity (only competition from multiple maters and unpaired guarders is assumed), so as seen in Figs. 9, 10, 11 and 12, increasing sex ratios mean the likelihood a given multiple-mating male will be successful decreases.

When the interbirth interval is low, females must provide care for a shorter period of time and are thus free to mate earlier. It also means that paired males do not have long to wait for the next paternity. With a larger population of free females, this results in a lower OSR, which corresponds to less competition for each additional paternity.

On the other hand, when birth intervals are long, paired males must wait longer for the next paternity. It also means that there are fewer free females in the population, resulting in a higher OSR, corresponding to an increased competition for each additional paternity.

## Conclusion

We have developed a mathematical model that allows us to explore the parameters that contributed to the evolution of monogamous behaviour and pair bonding. Our model is guided by the argument that connects the emergence of these pair bonds to the male-biased mating sex ratios that accompanied the evolution of human life history (Coxworth et al. 2015). We wish to use this model and subsequent models to help explain the differences between humans and our closest living relatives, the chimpanzees. This model does not assume anything about the details of male-male or male–female interactions, only their final outcomes as they relate to mean reproduction and mortality rates.

Additionally, there are many parameters that are known to trade off or have co-evolved with mating strategies. This includes many of the parameters that we assume are constant. However, the precise nature of these relationships are poorly understood and complex. By making a choice regarding the functional relationship between these various factors, we risk introducing unnecessary uncertainty and further complicating the analysis. Here, we deliberately take a simplifying approach to avoid any such choice. This perspective allows us to focus on fewer, more important parameters that play a bigger role in the dynamics of the problem.

The results indicate that scarcity of free, unpaired females relative to the number of unpaired males predicts the likelihood that guarding will be preferred over multiple mating. We’ve observed that this phenomenon is captured in both the ASR and OSR and consequently these ratios can be used to predict a shift in strategy.

The grandmother hypothesis suggests that subsidies from grandmothers to the offspring of their daughters shortened this interval; it was those subsidies that propelled the evolution of increased longevity without favouring continued female fertility to older ages (Hawkes et al. 1998; Hawkes 2020; Kim et al. 2019, 2014). In our model, this difference, along with greater longevity and longer-lasting pair bonds, allows mate-guarding to remain dominant in humans, which further supports (Coxworth et al. 2015). Nevertheless, this guarding dominance only holds if paternity uncertainty and the pair-bond break-up rate remain low.

Although we obtain intriguing results through analysis of this model, there remain limitations and questions. In our simulations, it is not obvious that chimps will adopt multiple mating while humans adopt mate guarding. This is likely a consequence of the simplifying assumptions of the model as no age structure is included. This can be further investigated by explicitly considering age structure, e.g., with an agent-based model. Additionally, direct patrilineal inheritance of strategy is unrealistic. It would be interesting to understand how a propensity to adopt a particular strategy is inherited or adapted to social and cultural influences within a lifetime. Also, how does male-male competition affect the outcome? The average age of first reproduction is female-biased (17 for females and 21 for males Marlowe 2004; Jones 2016). Even though the younger males are in the fertile ages, they do not get paternities because the older males out-compete them. This is a divergence from chimpanzee behaviour where adolescent males actually get paternities on the young females as the older males (who prefer older females Muller et al. 2006) do not prevent them Muller et al. (2020). Thus, there are important dynamics not captured by this model. Also, there may be other strategies males employ that affect the winning strategy at equilibrium. Guarding males may aid their offspring through provision of care and protection from infanticide, which is not included in this model.

Finally, how did guarding emerge in the first place? There is a long history of debate around how this may have evolved in our lineage and was likely influenced by a range of ecological and cultural factors. The predominant theory is that this behaviour evolved as a consequence of the benefits of cooperative parenting (Washburn and Lancaster 2017; Isaac 1978; Lovejoy 1981; Fisher 2016; Kaplan et al. 2000, 2010). One of the first models that explored alternative explanations found that there are broad conditions under which guarding became the dominant strategy (Hawkes et al. 1995). Our model provides support for the hypothesis that human pair bonds evolved with increasing payoffs for mate guarding, which resulted from the evolution of our grandmothering life history (Coxworth et al. 2015). However, there are many aspects of this process still left to be explored.

## Figures and Tables

**Fig. 15:**
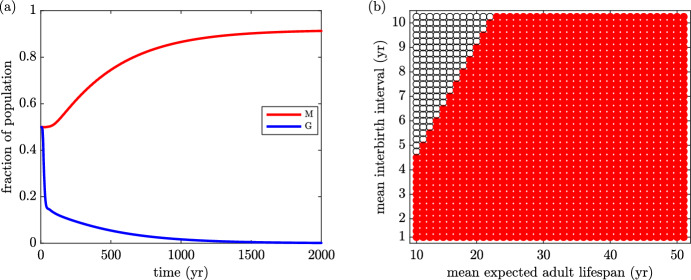
(Color Figure Online) Results when there is no end to the fertility period for females. **a** Trajectories for populations with average adult longevity $L=38$. In this case, there is no male-biased adult sex ratio and multiple-mating dominates. **b** The dominant strategy is multiple-mating for all values of expected adult lifespan, *L*, and the rate of offspring maturation, $\beta$. Notice in this case there is far less extinction as both *L* and $\beta$ increase since females can give birth up until death

## Data Availability

The authors confirm that the data supporting the findings of this study are included in the article. All relevant Matlab code and data for the analyses and results presented are available at github.com/mcnitschke/male mating ODE

## Derivation of the Density-Dependent Death Rate

In the main text, we use a density-dependent death rate $\mu (t)$, which contains two components:

22 $$\begin{aligned} \mu (t)=\frac{1}{L}+\mu _{variable}(t)\,, \end{aligned}$$

where the first term is a constant death rate given by the reciprocal of the individual’s expected adult life span, *L*, and the second term $\mu _{variable}(t)\ge 0$, is a variable density-dependent death rate. The constant component represents a minimal death rate that ensures each individual will have a maximum average expected adult lifespan of *L* regardless of the variable, density-dependent component.

To determine $\mu (t)$, we first assume $\mu (t)$ is such that the total adult population remains constant, which is possible when the growth rate exceeds the minimal death rate 1/*L*. Note this assumption implies the system has infinitely many equilibria, since any total initial adult population will remain constant. Nonetheless, this definition allows us to compare relative growth rates of the two male mating strategies without introducing new death-rate parameters.

Let $T_F = F^M + F^G + P_g^G+P^G+P_m^M+P_g^M+P^M + X + X_m + X_g$ be the total number of adult females and $T_M = M + G + P_g^G+P^G+P_m^M+P_g^M+P^M + Y$ be the total number of adult males. Since the total adult female and male populations remain constant,

$$\begin{aligned} \frac{dT_F}{dt} = \frac{dT_M}{dt} = 0. \end{aligned}$$

In addition, let us assume we start with an equal number of adult males and females, so that $$T_M = T_F = k $$ for some constant $$k > 0$$.

We now find the density-dependent death rate $\mu (t)$ in terms of other variables. We have

$$\begin{aligned} 0 = \frac{dT_M}{dt}&= \frac{dM}{dt} + \frac{dG}{dt} + \frac{dP_g}{dt} + \frac{dP}{dt} + \frac{dY}{dt} \\&= \frac{1}{2} \beta \big (F_m^M+F_g^G+F_m^G+F_g^M+P_g^G\\&\quad +P_m^M+P_g^M+X_g+X_m\big ) - \mu (t) T_M, \end{aligned}$$

so

$$\begin{aligned} \mu (t)&= \frac{\beta }{2k} \big (F_m^M+F_g^G+F_m^G+F_g^M+P_g^G+P_m^M+P_g^M+X_g+X_m\big ) , \end{aligned}$$

since $T_M = k$. We obtain the same expression if we use $dT_F/dt = 0$.

For simplicity, we can normalise all the population variables in (1)–(17) by *k*, so that the total male and female populations $T_M = T_F = 1$, and we are left with

$$\begin{aligned} \mu (t)&= \frac{\beta }{2} \big (F_m^M+F_g^G+F_m^G+F_g^M+P_g^G+P_m^M+P_g^M+X_g+X_m\big ) . \end{aligned}$$

Note we could go further and completely nondimensionalise the system by normalising time *t* by $1/\beta$, but since we will numerically simulate solutions, this step hardly reduces computational complexity and the resulting dimensionless parameters would be less convenient, since we intend to vary all life history parameters independently.

Hence, from (18), it follows that

$$\begin{aligned} \mu _{\text {variable}}(t) = \frac{\beta }{2} \big (F_m^M+F_g^G+F_m^G+F_g^M+P_g^G+P_m^M+P_g^M+X_g+X_m\big ) - \frac{1}{L}, \end{aligned}$$

whenever this expression is nonnegative, and $\mu _{\text {variable}}(t) = 0$ otherwise, so the expression for our density-dependent death rate is

23 $$\begin{aligned} \mu (t)&= \text {max} \left\{ \frac{1}{L}, \hspace{3mm} \frac{\beta }{2} \big (F_m^M+F_g^G+F_m^G+F_g^M+P_g^G+P_m^M+P_g^M+X_g+X_m\big ) \right\} . \end{aligned}$$

## The Case with no Female Fertility Loss

Figure 15 shows results when there is no end to female fertility. In this case, the multiple-mating strategy always dominates. The adult sex ratio is no longer male-biased, so less competition leads to multiple-mating behaviour. Additionally, notice that the interbirth interval has a smaller effect on extinction when *L* increases. If females are able to give birth throughout their entire lives, longer maturation periods do not lead to extinction.

## Detailed Description of Model Equations

Equation (1) models the population $F^M$ of free females carrying the multiple-mating trait without dependants. In the first term, free females conceive at rate $\rho$, causing them to enter the population $F_m^M$ of females with the multiple-mating trait caring for dependent offspring with the multiple-mating trait if they mated with a multiple-mating male, or evenly split between population $P_m^M$ and $P_g^M$ of pairs of guarding males and females caring for dependent offspring that either have the multiple-mating or guarding trait, if they mated with a guarding male.

In the second term, dependants reach independence at rate $\beta$, allowing them to enter the adult free-female population $F^M$ or the adult male multiple-mating population, *M*. All four populations caring for dependants with the multiple-mating trait ($F_m^M$, $F_m^G$, $P_m^M$, and $X_m$) contribute maturing dependants to the adult population at rate $\beta$. We include a factor of 1/2 because we assume half the offspring are female and half are male. For simplicity, we have left out a stage of juvenile independence between dependence and adulthood.

The third and fourth terms are the rates free females carrying the multiple-mating trait, who are caring for dependent offspring with the multiple-mating and guarding traits, return to the free-female population when their dependants reach independence at rate $\beta$ or die at rate $\delta _m$ or $\delta _g$. The fifth term is the rate at which paired females carrying the multiple-mating trait without dependants return to the free-female population when their pair bonds break up at rate $\chi$ or the male they are paired to dies at rate $\mu _M(t)$. The final term is the rate at which free females carrying the multiple-mating trait leave this category by either death at rate $\mu _F(t)$ or a loss of fertility at rate $\omega _F$.

Equation (2) models the population $F^G$ of free females carrying the guarding trait without dependants. In the first term, free females conceive at rate $\rho$, causing them to enter the population $P_g^G$ of pairs of guarding males and females carrying the guarding trait with dependent offspring that have the guarding trait if they mated with a guarding male, or evenly split between population $F_g^G$ and $F_m^G$ of females carrying the guarding trait caring for dependent offspring with either the guarding or multiple-mating trait if they mated with a multiple-mating male.

In the second term, dependants reach independence at rate $\beta$, allowing them to enter the adult free-female population $F^G$ or the adult male guarding population, *G*. All five populations caring for dependants with the guarding trait ($F_g^G$, $F_g^M$, $P_g^G$, $P_g^M$ and $X_g$) contribute maturing dependants to the adult population at rate $\beta$. We include a factor of 1/2 because we assume half the offspring are female and half are male. For simplicity, we have left out a stage of juvenile independence between dependence and adulthood.

The third and fourth terms are the rates free females carrying the guarding trait, who are caring for dependent offspring with the guarding and multiple-mating trait, return to the free-female population when their dependants reach independence at rate $\beta$ or die at rate $\delta _g$ or $\delta _m$. The fifth term is the rate at which paired females carrying the guarding trait without dependants return to the free-female population when their pair bonds break up at rate $\chi$ or the male they are paired to dies at rate $\mu _M(t)$. The final term is the rate at which free females carrying the guarding trait leave this category by either death at rate $\mu _F(t)$ or a loss of fertility at rate $\omega _F$.

Equation (3) models the population *M* of multiple-mating males. In the first term, maturing dependants enter the adult multiple-mating male population at rate $(1/2)\beta$ from the populations $F_m^M$, $F_m^G$, $P_m^M$ and $X_m$ of fertile and post-fertile females caring for dependent offspring carrying the multiple-mating trait. As before, we include a factor of 1/2 because we assume half the offspring are male. The second term is the rate multiple-mating males gain dependants by stealing paternities from guarding males. Here, *q* is the level of paternity uncertainty, or the probability a dependant of a guarding male is actually the offspring of a multiple-mating male. We assume paired guarding males are unaware of paternity thefts, so the theft is only accounted in the model when dependants mature. Consequently, males may remain paired to females caring offspring of multiple-mating males. As before, $\beta$ is the rate dependants mature, the factor of 1/2 accounts for the fraction of offspring who are male, and $F_g^G$, $F_g^M$, $P_g^G$, $P_g^M$, and $X_g$ are the five populations caring for dependants of guarding males. In the final term, multiple-mating males stop competing for mates due to frailty at rate $\omega _M$ and die at the population-density-dependent death rate $\mu _M(t)$.

Equation (4) models the population *G* of guarding males. The first term is the rate guarding males productively mate with free females. We assume productive matings occur at rate $\rho (F^M+F^G)$, which is the overall conception rate of the full female population. Then, for simplicity, we assume females do not favour guarding males or multiple-mating males, so the proportion of paternities going to guarding males is simply the frequency $G/(M+G)$ of guarding males in the available, unpaired male population. The second term is the rate maturing dependants enter the adult guarding-male population from the populations $F_g^G$, $F_g^M$, $P_g^G$, $P_g^M$, and $X_g$ with dependent offspring carrying the guarding trait. It is the counterpart to the second term of (3), and the factor $1-q$ is the probability a dependant of a guarding male is his offspring. The third term is the rate paired males return to unpaired guarding population due to pair-bond break-up at rate $\chi$, female mates’ fertility ending at rate $\omega _F$, which we assume dissolves the pair, or female mates dying at rate $\mu _F(t)$. We assume that if females with dependants die, their dependants die too, so unpaired males do not transition to caring for any remaining dependants. In the final term, guarding males stop competing for mates due to frailty at rate $\omega _M$ and die at rate $\mu _M(t)$.

Equation (5) models the population $F_m^M$ of females caring for dependent offspring with the multiple-mating trait who are carrying the multile-mating trait themselves. The first term is the rate free females conceive with multiple-mating males. This rate is the product of the overall female conception rate $\rho F^M$ multiplied by the frequency $M/(M+G)$ of multiple-mating males. In the second term, paired females carrying the multiple-mating trait who are caring for dependent offspring with the multiple-mating trait return to the unpaired female population when either they break up with their partner at rate $\chi$ or the male partner dies at rate $\mu _M(t)$. In the final term, females leave this population when either their dependants mature at rate $\beta$, dependants die at rate $\delta _m$, the female experiences fertility loss at rate $\omega _F$, or the female dies at rate $\mu _F(t)$.

Equation (6) models the population $F_g^G$ of females caring for dependent offspring with the guarding trait who are carrying the guarding trait themselves. The first term is the rate free females conceive with multiple-mating males. This rate is the product of the female conception rate for females carrying the guarding trait, $\rho F^G$, multiplied by the frequency $M/(M+G)$ of multiple-mating males. We include a factor of 1/2 because we assume that when females carrying the guarding trait mate with a multiple-mating male, half of the offspring inherit the multiple-mating trait and the other half inherit the guarding trait. In the second term, paired females carrying the guarding trait who are caring for dependent offspring with the guarding trait return to the unpaired female population when either they break up with their partner at rate $\chi$ or the male partner dies at rate $\mu _M(t)$. In the final term, females leave this population when their dependants mature at rate $\beta$, dependants die at rate $\delta _g$, the female experiences fertility loss at rate $\omega _F$, or the female dies at rate $\mu _F(t)$.

Equation (7) models the population $F_m^G$ of females caring for dependent offspring with the multiple-mating trait who are carrying the guarding trait. The first term is the rate free females conceive with multiple-mating males. This rate is the product of the female conception rate for females carrying the guarding trait, $\rho F^G$, multiplied by the frequency $M/(M+G)$ of multiple-mating males. We include a factor of 1/2 because we assume that when females carrying the guarding trait mate with a multiple-mating male, half of the offspring inherit the multiple-mating trait and the other half inherit the guarding trait. In the final term, females leave this population when their dependants mature at rate $\beta$, dependants die at rate $\delta _m$, the female experiences fertility loss at rate $\omega _F$, or the female dies at rate $\mu _F(t)$.

Equation (8) models the population $F_g^M$ of females caring for dependent offspring with the guarding trait who are carrying the multiple-mating trait. In the first term, paired females carrying the multiple-mating trait who are caring for dependent offspring with the guarding trait return to the unpaired female population when either they break up with their partner at rate $\chi$ or the male partner dies at rate $\mu _M(t)$. In the final term, females leave this population when their dependants mature at rate $\beta$, dependants die at rate $\delta _g$, the female experiences fertility loss at rate $\omega _F$, or the female dies at rate $\mu _F(t)$.

Equation (9) models the population $P_g^G$ of pairs of guarding males and females carrying the guarding trait who are caring for dependent offspring with the guarding trait. The first term is the rate free females conceive with guarding males, causing them to have dependants and form pairs. This rate is the product of the female conception rate for females carrying the guarding trait, $\rho F^G$ multiplied by the frequency $G/(M+G)$ of guarding males. The second term is the rate male–female pairs without dependants conceive at rate $\rho$ and enter the population of pairs with dependants. The final term is the rate at which males and females leave this category which happens if their dependent offspring mature at rate $\beta$, their dependent offspring die at rate $\delta _g$, the male partner dies at rate $\mu _M(t)$, or the female partner dies at rate $\mu _F(t)$.

Equation (10) models the population, $P^G$, of pairs of guarding males and females carrying the guarding trait, without dependants. The first term is the rate paired females and guarding males conceive at rate $\rho$, causing them to enter the population $P_g^G$. The second term is the rate dependants mature at rate $\beta$ or die at rate $\delta _g$, causing pairs in $P_g^G$ to enter population $P^G$. The third term is the rate pairs dissolve due to pair-bond break-up at rate $\chi$, females losing fertility at rate $\omega _F$, or either mate dying at rate $\mu _M(t)$ or $\mu _F(t)$.

Equation (11) models the population $P_m^M$ of pairs of guarding males and females carrying the multiple-mating trait who are caring for dependants with the multiple-mating trait. The first term is the rate free females conceive with guarding males, causing them to have dependants and form pairs. This rate is the product of the female conception rate for females carrying the multiple-mating trait, $\rho F^M$ multiplied by the frequency $G/(M+G)$ of guarding males. We include a factor of 1/2 because we assume that when females carrying the multiple-mating trait mate with a guarding male, half of the offspring inherit the multiple-mating trait and half inherit the guarding trait. The second term is the rate male–female pairs without dependants conceive at rate $\rho$ and enter the population of pairs with dependants. The extra factor of 1/2 is included for the same reason concerning the previous term. The final term is the rate at which males and females leave this category which happens if their dependants mature at rate $\beta$, their dependants die at rate $\delta _m$, the pair breaks up at rate $\chi$, the male partner dies at rate $\mu _M(t)$, or the female partner dies at rate $\mu _F(t)$.

Equation (12) models the population $P_g^M$ of pairs of guarding males and females carrying the multiple-mating trait who are caring for dependants with the multiple-mating trait. The first term is the rate free females conceive with guarding males, causing them to have dependants and form pairs. This rate is the product of the female conception rate for females carrying the multiple-mating trait, $\rho F^M$ multiplied by the frequency $G/(M+G)$ of guarding males. We include a factor of 1/2 because we assume that when females carrying the multiple-mating trait mate with a guarding male, half of the offspring inherit the multiple-mating trait and half inherit the guarding trait. The second term is the rate male–female pairs without dependants conceive at rate $\rho$ and enter the population of pairs with dependants. The extra factor of 1/2 is included for the same reason concerning the previous term. The final term is the rate at which males and females leave this category which happens if their dependants mature at rate $\beta$, their dependants die at rate $\delta _g$, the pair breaks up at rate $\chi$, the male partner dies at rate $\mu _M(t)$, or the female partner dies at rate $\mu _F(t)$.

Equation (13) models the population, $P^M$, of pairs of guarding males and females carrying the multiple-mating trait, without dependants. The first term is the rate paired females and guarding males conceive at rate $\rho$, causing them to enter the population $P_m^M$ or $P_g^M$ with equal probability. The second term is the rate pairs in $P_m^M$ enter the population $P^M$ when dependants mature at rate $\beta$ or die at rate $\delta _m$. The third term is the rate pairs in $P_g^M$ enter the population $P^M$ when dependants mature at rate $\beta$ or die at rate $\delta _g$. The final term is the rate pairs dissolve due to pair-bond break-up at rate $\chi$, females losing fertility at rate $\omega _F$, or either mate dying at rate $\mu _M(t)$ or $\mu _F(t)$.

Equation (14) models the population *X* of post-fertile females without dependants. The first term is the rate free females in $F^M$, $F^G$ and paired females in $P^M$, $P^G$ end fertility at rate $\omega _F$, causing them to enter *X*. The second and third terms are the rates dependants mature at rate $\beta$ and die at rate $\delta _m$ or $\delta _g$, causing post-fertile females in $X_m$ and $X_g$ to enter *X*. The final term is the rate females in *X* die at rate $\mu _F(t)$.

Equation (15) models the population $X_g$ of post-fertile females caring for dependent offspring with the guarding trait. The first term is the rate paired and unpaired fertile females caring for dependent offspring with the guarding trait lose fertility at rate $\omega _F$, causing them to enter $X_g$. The second term is the rate dependants mature at rate $\beta$ or die at rate $\delta _g$, causing females to leave $X_g$ and enter the post-fertile population without dependants. The final term is the rate females in $X_g$ die at rate $\mu _F(t)$.

Equation (16) models the population $X_m$ of post-fertile females caring for dependent offspring with the multiple-mating trait. The first term is the rate fertile females caring for dependent offspring with the multiple-mating trait end fertility at rate $\omega _F$, causing them to enter $X_m$. The second term is the rate dependants mature at rate $\beta$ or die at rate $\delta _m$, causing females to leave $X_m$ and enter the post-fertile population without dependants. The final term is the rate females in $X_m$ die at rate $\mu _F(t)$.

Equation (17) models the population *Y* of males who no longer compete due to frailty. The first term is the rate unpaired males in *M* and *G* become frail at rate $\omega _M$, causing them to enter this post-fertile population *Y*. Paired males who enter frailty remain in the pair until it ends but no longer seek additional mates afterwards. The final term is the rate males in *Y* die at rate $\mu _M(t)$.
